# Neurophysiological correlates of various mental perspectives

**DOI:** 10.3389/fnhum.2014.00637

**Published:** 2014-08-21

**Authors:** Thilo Hinterberger, Milena Zlabinger, Klaus Blaser

**Affiliations:** ^1^Section of Applied Consciousness Sciences, Department of Psychosomatic Medicine, University Medical Center RegensburgRegensburg, Germany; ^2^Brain, Mind and Healing Program, Samueli InstituteAlexandria, VA, USA; ^3^Department of Psychology, University of Tübingen TübingenGermany; ^4^Center for Applied Boundary StudiesBasel, Switzerland

**Keywords:** mental perspectives, attentional focus, EEG, intrapersonal space, empathy

## Abstract

A common view of consciousness is that our mind presents emotions, experiences, and images in an internal mental (re-)presentation space which in a state of wakefulness is triggered by the world outside. Consciousness can be defined as the observation of this inner mental space. We propose a new model, in which the state of the conscious observer is defined by the observer’s mental position and focus of attention. The mental position of the observer can either be within the mental self (intrapersonal space), in the mental outer world (extrapersonal space) or in an empathic connection, i.e., within the intrapersonal space of another person (perspective taking). The focus of attention can be directed toward the self or toward the outside world. This mental space model can help us to understand the patterns of relationships and interactions with other persons as they occur in social life. To investigate the neurophysiological correlates and discriminability of the different mental states, we conducted an EEG experiment measuring the brain activity of 16 subjects via 64 electrodes while they engaged in different mental positions (intrapersonal, extrapersonal, perspective taking) with different attentional foci (self, object). Compared to external mental locations, internal ones showed significantly increased alpha2 power, especially when the observer was focusing on an object. Alpha2 and beta2 were increased in the empathic condition compared to the extrapersonal perspective. Delta power was significantly higher when the attentional focus was directed toward an object in comparison to the participant’s own self. This exploratory study demonstrates highly significant differences between various mental locations and foci, suggesting that the proposed categories of mental location and intra- and interpersonal attentional foci are not only helpful theoretical concepts but are also physiologically relevant and therefore may relate to basic brain processing mechanisms.

## INTRODUCTION

Continuously and with increasing interest, the scientific fields of philosophy and neuroscience are concentrating on the study of the phenomenon of consciousness. Research on altered states of consciousness, meditation, sleep, and out-of-body experiences has become popular in the scientific community. However, there is still a lack of understanding the links between consciousness as a first-person experience and the variety of related psychophysiological results. One of the most challenging problems arises from the categorical incongruences between the concepts of subjective mental experience and the physiological description of the brain. Efforts to approach the problem have been made by Damasio (1999) and Metzinger (2009), among others. Physiological measurements can also be used to justify psychological concepts if their physiological correlates discriminate those concepts. The aim of the present study was to contribute to a new mental model with electrophysiological data as correlates. This model was termed Boundary-Based Awareness Model (BBAM; Blaser, 2011, 2012, 2013) and assumes a structure for the relationship between the observer and the observed mental (re-)presentation that distinguishes between various mental positions of the observer as follows: (1) the inner self model, (2) the physical world model, and (3) empathic connection with other individuals. The model further distinguishes between the corresponding attentional foci. Although the BBAM has been confirmed by two questionnaires, namely the boundary protection scale (BPS; Blaser et al., 2014b) and the interpersonal attention management inventory (IAMI; Blaser et al., 2014a), the physiological role of these mental perspectives has never been studied before. This was the focus of the study presented here. As a first step, this study is an exploratory approach that might generate hypotheses, but it was not designed to give clear evidence of the assumed underlying processes.

### PHYSICAL AND MENTAL WORLD

Fundamentally, consciousness is a phenomenon that occurs in a subjective mental domain. It may be regarded as the system-immanent view of neuronal information processing. Therefore, to better understand the intriguing question of how we perceive and understand the physical world and also another person’s mind, it is helpful to presume the model of an inner space of the mind and an inner self. Early on, James (1892) distinguished between different kinds of self, such as the physical self, the mental self and the spiritual self. These distinctions seem to reappear in recent concepts of self as discussed in neuroscience (Panksepp, 1998; Damasio, 1999; Gallagher, 2000; Churchland, 2002; Kelley et al., 2002; Turk et al., 2003; Vogeley and Fink, 2003; Dalgleish, 2004; Northoff and Bermpohl, 2004). In modern neuroscience and neurophilosophy, a common view is that our mind represents emotions, experiences and images in an internal mental (re-)presentation space, which in a state of wakefulness is triggered by the outside world (Damasio, 1999; Blaser, 2008; Metzinger, 2009; Hinterberger, 2011). The sensory system can be seen as the physical interface which enables us to come into contact with objects, events and even the emotional contents of other people apart from us. Sensory information becomes entangled with the current mind state, creating the present experience within an inner mental space. For example, the interaction of the mental self with the mental outside world plays a crucial role in the new understanding of schizophrenia (Taylor, 2011). However, using this model for consciousness, it should be noted that being conscious is not just related to the existence of such an inner representation. Moreover, consciousness requires an observer and therefore can be defined as the observation of this inner mental space. While the observer him/herself remains abstract, the self is represented in such a spatial model as the self-model as described by Damasio (1999) or Metzinger (2009). The self can be thought of as part of the inner representation space, more or less separated from the world model that carries those observed objects which are assessed as being separate from our own body. In fact the concept of the self involves a number of different brain functions depending on whether we speak of a biographical self, the cognitive image of the self or the self as an embodied sensory perception. In this context we do not differentiate those aspects because they all might be present to some extent when a person is asked to observe him/herself.

### SELF AND OTHER

The mental self can be thought of as embedded in an interconnected fashion within the mental representation space (Damasio, 1999; Northoff et al., 2006). Thus, in analogy to the physical body in which the skin is the boundary that separates us from the outside world, a mental boundary can be attributed to the mental self. Psychotherapists already work with this model and often share a common understanding when speaking about thick and thin boundaries (Tausk, 1992). Accordingly, a protective mental boundary would mean that our mental self is clearly separated from the mental outer world, while in individuals with a blurred boundary, the self and the outer world may overlap and sometimes cannot be clearly distinguished from each other.

The outer world does not only contain more or less meaningful objects. Moreover, social life occurs in this realm, and therefore the mental world model is filled with representatives and concepts of other people one knows. This could explain why therapists often realize that people with thin boundaries sometimes have difficulties with the distinction between emotions, feelings and thoughts belonging to them and those of another person. The Boundary Protection Scale (BPS-20) is a psychometric instrument for determining the properties of an individual’s mental boundary (Blaser et al., 2014b). The ability to read the mental states of a fellow human is called mentalizing (Fonagy et al., 2002; Allen et al., 2008). The neural basis of mentalizing and how we distinguish between the self and the other has been studied by many authors (Decety and Sommerville, 2003; Northoff and Bermpohl, 2004; Frith and Frith, 2006a,b; Uddin et al., 2007; Castiello et al., 2010). Special ways of understanding others are represented by compassion (Gusnard et al., 2001; Goetz et al., 2010; Klimecki et al., 2013), empathy (Singer, 2006; Lamm et al., 2007; Hooker et al., 2008; Singer and Lamm, 2009) and theory of mind (Gallese and Goldman, 1998; Vogeley, 2001; Gallagher and Frith, 2003; Völlm et al., 2006). They are, as will be described later on, expressions of different mental perspectives toward the self and the other.

### CONSCIOUS OBSERVATION/MENTAL FOCUS AND LOCATION

In this model the “conscious observer” in us is neither identical with the self, nor is it a part of the world, but its mental representation can be attached to or even identified with one of them. In the spatial model of consciousness described above, at least two properties can be attributed to the conscious observer, thus defining his/her state: the mental position or location and the focus or direction of attention^1^. Those properties define the mental viewpoint or perspective.

According to our model, there are at least three places in which the observer can be located: (1) within and in connection with the mental self-construct, which we call the *intrapersonal space* or internal self-referential perspective, (2) within the outer world, free and independent in the mental space which we call the *extrapersonal space* or external perspective, and (3) *perspective taking,* i.e., an empathic connection to another person which can be either cognitive or affectively embodied (Blaser, 2008, 2012, 2013). Accordingly, the focus of attention can be directed either toward the self, i.e., the intrapersonal space, or toward the world, i.e., the extrapersonal space or another person within the extrapersonal space. Both the mental position and focus of attention define the mental perspective of a conscious observation. The combinations of the various mental locations and foci result in ten different perspectives, listed in **Table 1**. They represent the modes postulated by the BBAM (Blaser, 2011, 2012, 2013). To test the spatial attention model on another level, the authors developed a questionnaire, the IAMI. The validation of this new self-rating instrument confirms the concept of an intrapersonal space, an extrapersonal space and the extrapersonal mental space of a fellow human. The IAMI constitutes a tool for assessment of the ability to manage the various states in daily life (Blaser et al., 2014a; see Materials and Methods).

**Table 1:** Overview of mental localization, attentional focus, and processing modality according to the BBAM.

No.	Mode	Mental location	Attentional focus	Processing modality (cognitive or affective)
1	IS	Intrapersonal	Own self	Affective (mindful self-centered interoception)
2	IF	Intrapersonal	Object in the outer world	Affective (mindful)
3	IP	Intrapersonal	Another person whose mental location is inside his/her intrapersonal space	Affective (compassion)
4	OS	Extrapersonal	Own self	Cognitive self-perception
5	OF	Extrapersonal	Object in the outer world	Cognitive
6	E_cog_F	Perspective taking	Object in the outer world via another person whose mental location is in the outer world	Cognitive (theory of mind)
7	E_cog_P	Perspective taking	Another person whose mental location is inside his/her intrapersonal space	Cognitive (theory–theory)
8	ES	Perspective taking	Own self	Affective (empathy)
9	E_aff_F	Perspective taking	Object in the outer world	Affective (empathy)
10	E_aff_P	Perspective taking	Another person in his/her intrapersonal space	Affective (empathy)

*The first letter in the mode designation codes the mental location while the second letter codes the attentional focus. Some states are perceived more affectively while others are perceived more cognitively.*

*Mental locations: I, intrapersonal; O, extrapersonal; E, perspective taking (empathic). Attentional foci: S, self; F, object outside of the self; P, other person.*

With this mental space model and its interpersonal framework we attempt to reduce mindfulness, compassion, cognitive self-perception, theory of mind, theory–theory and empathy to a common denominator (Blaser, 2012). This novel theoretical framework weaves mental life and interpersonal dynamics together. It enables us to understand the patterns of relationships and what occurs when we come into contact with another person.

### NEUROSCIENTIFIC CONCEPTS

Although we do not intend to discuss all the neuroscientific aspects of these concepts we do aim to present some neuroscientific findings relating to the proposed concepts.

The discriminability of mental foci has been shown previously in studies on self-perception, for example the involvement of cortical midline structures in self-reference as seen by neuroimaging studies (Northoff and Bermpohl, 2004).

In earlier research it could be shown that the first-person perspective and the third-person perspective (comparable with the mental locations “intrapersonal space” and “external intrapersonal space” in our model) rely on differential neural processes. For example, Vogeley et al. (2004) were able to identify different brain regions activated by observation tasks when researching mental states of the first- versus the third-person perspective), while Ruby and Decety (2001) used PET measurements to explore the cognitive and neural processing involved in agency.

Some of the modes listed in **Table 1** often occur in the resting state, i.e., while no external task that demands a large amount of resources from the brain functions is being performed. During resting conditions, a person’s mind is usually engaged in information processing, memorization, self-referential thoughts, evaluations, etc. All these tasks require the activation of so-called resting state networks in the brain. One of them is the default mode network (DMN; Raichle et al., 2001), which comprises a number of non-goal-oriented mental processing functions such as task-independent introspection or self-referential thoughts. From the literature we could not decide whether the DMN relates more to a self-directed attentional focus or an embodied self-centered mental location. Therefore, one could expect the DMN to be active during the intrapersonal modes but also in self-directed modes.

In this study we focus on the correlates with EEG data. This necessitates an introduction to some related concepts with the corresponding EEG results.

#### Intrapersonal space (I)

Jann et al. (2010) and Mantini et al. (2007) described EEG correlates of the resting state networks. They found that the DMN, which we would assume to be active in intrapersonal mental locations, correlated with increased frontocentral alpha1, posterior, and occipital alpha2 and parietal beta1. Delta and theta were decreased. Ward (2003) also reports that alpha could increase during attentional tasks in order to avoid distraction, and alpha has also been shown to be stronger when attention was directed toward internal mental imagery rather than external input (Cooper et al., 2003).

#### Extrapersonal space (O)

In contrast, the extrapersonal modes require adoption of an external viewpoint and might therefore reduce DMN activity.

#### Perspective taking (E)

Perspective taking or imagination of a well-known person is a memory-related task. Increased theta activity has been found to be associated with memory functions, i.e., both encoding and retrieval of information (Klimesch, 1999; Başar et al., 2000; Ward, 2003).

#### Attentional focus

A self-directed mental focus could also be part of the DMN (Knyazev, 2013), leading to decreased delta and theta band activity and increased frontocentral alpha1, posterior, and occipital alpha2 and parietal beta1 (Mantini et al., 2007; Jann et al., 2010).

#### Cognitive versus affective processing

As listed in **Table 1** some of the intrapersonal modes are attributed to emotional and affective processing, i.e., all perceptions are related to the person’s own physical being. In contrast the extrapersonal modes are attributed to cognitive processing as all perceptions (of the self, of other persons or of external objects) are cognitively evaluated. Perspective taking can be cognitive (theory of mind or theory–theory), but if it occurs with an empathic attitude and emotions are involved, we treat it as affective. Electrophysiologically, cognitive processing is associated with a decrease in alpha band activity and an increase in theta activations (e.g., Ramos et al., 1993; Klimesch, 1996, 1999). Ray and Cole (1985) attributed alpha to the attentional aspect and beta waves to cognitive and emotional processing, with activation in the temporal areas for emotionally positive or negative tasks and in the parietal areas for cognitive tasks. The prefrontal sector is most directly associated with emotion (for an overview and commentary see Davidson, 2004). There is no consistent pattern of alpha activity comparing neutral and affective stimuli. Aftanas et al. (2002, 2004), for example, found an increase in posterior and anterior alpha with affective stimuli, whereas De Cesarei and Codispoti (2011) found a decrease in posterior sites. Uusberg et al. (2013) found enhanced high alpha in central and parietal areas in late event-related potentials with emotional stimuli, most prominently with aversive stimuli. Müller et al. (1999) identified the temporal areas as being associated with positive (right hemisphere) and negative (left hemisphere) emotions. Regardless of the valence they found enhanced gamma band power (30–50 Hz) at right frontal electrodes with emotional processing compared to processing of neutral pictures. Although the field of cognitive and affective neuroscience is large most studies have been performed with visual stimuli, and a clear correspondent of our tasks could not be found in the literature. This restricts the formulation of a very specific hypothesis.

More detailed relationships between these concepts and the states examined in our study will be mentioned in the section “Materials and Methods.” An explicit classification of mental location, mental foci and their combinations has never been studied before. However, as these concepts overlap with well-researched concepts it can be supposed that the modes of the model presented here discriminate between each other as well.

The aim of the present study was to assess EEG pattern differences between mental location and the direction of the attentional focus. The existence of neurophysiological differences between the mental states defined in the spatial attention concept would demonstrate as a first step the neurophysiological relevance of this concept. Our hypothesis in this study was that in a guided exercise the various tasks would not only represent subjectively different states of consciousness but also show significantly different patterns on spectral EEG data. This was tested by measuring 64 channels of EEG in 16 participants who were guided through six of the mental states from **Table 1** to investigate the differences between mental locations and also between attentional foci. A more specific formulation of the hypothesis is given in the section “Materials and Methods.”

## MATERIALS AND METHODS

### PARTICIPANTS

Sixteen healthy participants (10 female, 6 male) aged between 33 and 70 years (mean: 52 years) took part in one experimental session and gave written informed consent. All of them had previously participated in a workshop in which they had practiced the different mental states through the IAMEx as presented below. To verify that the studied sample showed no pathological conspicuity but rather increased mental abilities all participants completed the following questionnaires: the IAMI, BPS-20, Revised Symptom Check List (SCL-90R), and freiburg mindfulness inventory (FMI). The study was approved by the legal ethics committee of the University Clinic Regensburg.

### STUDY DESIGN

#### Assessment through questionnaires

***Boundary Protection Scale (BPS-20).*** Blaser constructed the BPS-20 to assess the ability of a person to maintain his/her personal boundaries. It consists of 20 items, six of them framed negatively, which are summed to yield a single boundary protection value. The higher this value, the worse is the boundary protection. Ratings are given on a scale from 1 (almost never) to 5 (almost always). The internal consistency of this scale was found to be 0.71 (Cronbach’s alpha) in a validation study with 1,089 participants (Blaser et al., 2014b).

***Interpersonal attention management inventory (IAMI).*** This consists of 50 items dealing with everyday life experiences leading to 10 factor items assessing the ability to control the direction of the attentional focus and the mental location. As each of the 10 subfactors is related to one of three mental locations it is possible to construct three major factors assessing the ability to manage the inner, the outer and the empathic mental perspective. Ratings are given on a scale from 1 (almost never) to 5 (almost always). The IAMI was validated in the same study as the BPS-20 involving 1,089 participants and showed an internal consistency of 0.87 (Cronbach’s alpha; Blaser et al., 2014a).

***Freiburg Mindfulness Inventory (FMI).*** Mindfulness, with its subfactors *presence* and *acceptance,* was measured using the 14-item version of the FMI (Buchheld et al., 2001; Buchheld and Walach, 2002; Walach et al., 2006). The FMI assesses self-ratings of awareness and non-judgment of present-moment experiences (Buchheld et al., 2001; Buchheld and Walach, 2002; Heidenreich et al., 2006; Walach et al., 2006; Kohls et al., 2009). Sample items are “I am open to the experience of the present moment” and “I accept unpleasant experiences.” Ratings are given on a scale from 1 (no, never) to 4 (yes, always). The global FMI scale yielded an internal consistency of 0.83 (Cronbach’s alpha), while the two subfactors only reached 0.71 (presence) and 0.64 (acceptance; Kohls et al., 2009). The validation study with 1,089 participants showed an internal consistency of 0.83 (Cronbach’s alpha) for the FMI.

***Revised Symptom Check List (SCL-90R).*** Psychopathological symptoms were assessed using the SCL-90R self-rating questionnaire (Franke, 2002) with 90 items focusing on 9 scales. Additionally, three global factors can be calculated, namely a Global Severity Index, the Positive Symptom Distress Index and the Positive Symptom Total. In our analysis, all of the values were transformed into normalized *t*-values with a mean of 50 and a standard deviation of 10. Cronbach’s alpha was reported to be in the range between 0.75 and 0.97.

#### The Interpersonal Attention Management Exercise (IAMEx)

**FIGURE 1:**
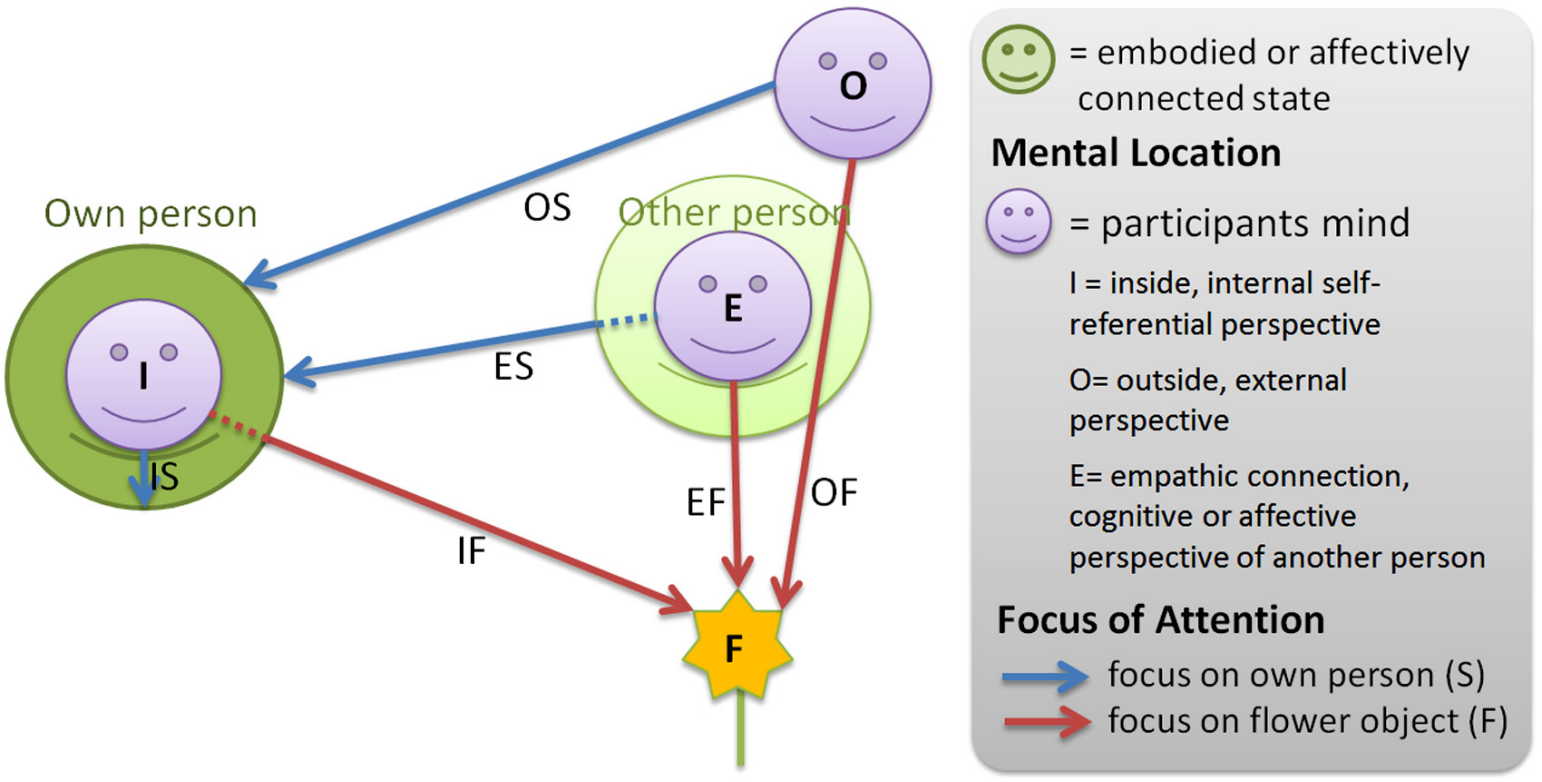
**Illustration of the 6 task conditions in this study**. Participants were asked to adopt three different mental locations in which they focused either on their own body or on flowers in the room. The states are identified by two-letter acronyms describing location and focus.

For therapeutic treatment Blaser has developed a mental exercise termed the IAMEx. The IAMEx is used to train voluntary achievement of the various states and representation modes. This exercise was practiced with all participants and comprised the main part of this study. It provided the various mental states for the neurophysiological discrimination of different mental locations, attentional foci and processing modes. For the purposes of experimental consolidation we restricted our study to the mental foci self and object. Therefore, only six of the mental perspectives taken from the list in **Table 1** were studied physiologically by EEG measurements during the instructed exercise. Explicitly, in our study the participants were guided through the states displayed in **Figure 1**, characterized as follows:

***Mode IS.*** This involves directing the focus of one’s attention at the internal from within the intrapersonal space (self-centered introspection). The bodily sensation is the experience of being present in the moment of subjective experience as it occurs (Stern, 2004). This also relates to so-called focusing, a method that enables a person to get in touch with his/her emotions physically through bodily experience (Gendlin, 1998). This is related to the theory of Damasio (1999), who coined the phrase “I feel, therefore I am.”

*Instruction.* Try to center yourself. Perceive your breath. Are you breathing deeply or superficially, slow or fast, with the chest, with the belly or with both? There is no need for you to change anything, only to perceive. Are you aware of any feelings and do you notice bodily sensations? Where do you perceive something? Yield to it.

***Mode IF.*** Directing the attention from the intrapersonal space to the exterior world, one is still connected to the physical body in a mindful way. To look outside from within involves a bi-focussed mode of perception, i.e., perceiving the outer world and simultaneously feeling one’s own bodily sensations. This mental state is central to mindfulness-based therapy forms (Segal et al., 2002).

*Instruction.* Now turn your attention to the flowers (which stand on a table 3 m away from the participant). Remain self-centered and mindful. What changes occur in your bodily sensations when you perceive the plants mindfully?

***Mode OS.*** By crossing one’s own self-boundary with the location of attention from inside to outside one arrives in a cognitive mental state, disconnected from the inner world and bodily sensations; functions such as the working memory, problem solving, analyzing, and planning of processes are activated. Simultaneously, the DMN activity should be reduced. That enables cognitive self-perception, i.e., to look from the outside, from a meta-position, at one’s own feelings. It is associated with the normal forms of dissociation in contrast to the pathological forms (Putnam, 1997). Previc (2009, 2011) associates distant extrapersonal states such as dreaming, hallucinations, out-of-body experiences or religious activity with ventral dopaminergic pathways.

*Instruction.* Imagine you’re standing on a white sheet of paper which lies 4 m in front of you, looking from there to yourself sitting on the chair with the electrode cap on your head. What are you seeing? How is this for you?

***Mode OF.*** As with mode OS the mental location is in the extrapersonal space, but the focus of attention is an object in the outer world. This is associated with cognitive control, for example as described by Herwig et al. (2007), which is important for an objective viewpoint toward an object in the outer world. The intended state does not require a self and is therefore called a “selfless”^2^ state.

*Instruction.* Imagine you’re still standing on the white sheet of paper, now looking from there to the flowers. How is it now, when you’re looking from there to the flowers?

***Mode EF.*** As with modes OS and OF the mental location is in the extrapersonal space, but the focus of attention are the thoughts of another person about an object. This is known as the theory of mind or cognitive empathy (Shamay-Tsoory et al., 2009) in the sense of thinking about the thoughts of another person (Gopnik and Wellman, 1992).

*Instruction.* Imagine a previously determined person now enters the room and positions herself to the left of you on a green sheet of paper. What is she thinking, when she is looking at the flowers from there? What do you guess she is thinking looking at the flowers?

***Mode ES.*** With one’s mental location in an external intrapersonal space and the focus on one’s own person, one can look in an empathic way at one’s own feelings. In contrast to cognitive empathy and the mirror neuron system, which displays a form of affective or emotional empathy (Shamay-Tsoory et al., 2009), for some authors this is (together with modes E_aff_F and E_aff_P, see **Table 1**) the “real” form of empathy, whereas the other forms (modes IP, E_cog_F and E_cog_P, see **Table 1**) are not denoted with the term “empathy” (e.g., Singer and Lamm, 2009). This can be described as looking through the emotional glasses of another person and can be achieved by asking the question “how would it be for me?” (Lamm et al., 2007).

*Instruction.* Imagine the person takes one step away from you and you take her place on the green sheet of paper and empathize with her. What is she feeling, when she looks from there at you sitting on the chair with the electrode cap? What are your bodily sensations when looking through her emotional glasses at yourself sitting there?

#### Experimental procedure

A sequence of nine different instructions led the participant through all states of interest within one IAMEx as listed in **Table 2**. The experimental session was subdivided into three EEG recording phases. The first phase consisted of a short reference measurement with 2 min of the participant sitting relaxed with open eyes, 2 min of sitting with closed eyes and another 2 min of reading a text from an arbitrary book. Recording phases 2 and 3 were carried out with open eyes. Each of them consisted of a guided IAMEx according to **Table 2**. After each instruction the corresponding state should be maintained for about 1 min, so the whole exercise took a bit less than 15 min including the time for the instructions. Recording phase 3 repeated phase 2 in order to increase statistical power and possibly test for retest stability. After both phases 2 and 3 the participant was asked on a self-rating scale how well he/she was able to fulfill each of the experimental tasks 2–8 in **Table 2**.

**Table 2:** Sequence of different tasks in the IAMEx.

No.	Task	Mental location	Attentional focus	Processing modality
1	Resting with eyes open	U	U	U
2	Intrapersonal mental location observing the self from within and one’s own body (mindful interoception, self-centered state)	I	S	A
3	Intrapersonal mental location observing an object in the outer environment	I	F	A
	Leaving the inner boundary			
4	Extrapersonal mental location observing the self from an outside viewpoint (cognitive self-perception)	O	S	C
5	Extrapersonal mental location, observing an object in the outer environment (“selfless” state)	O	F	C
6	Taking over the cognitive perspective of another person and observing the same object (theory of mind)	E	F	C
7	Merging with another person observing the self and physical sensations (empathic self-perception)	E	S	A
	Approaching the self and reconnecting			
8	Intrapersonal mental location experiencing one’s own physical sensations and observing the self (mindful interoception)	I	S	A
9	Resting with eyes open	U	U	U

*The right columns indicate the mental location and the focus which define a task.*

*Location: U, undefined; I, inside; O, outside; E, perspective taking (empathic).*

*Focus: U, undefined; S, self or own physical sensations; F = flowers.*

*Processing modality: U, undefined; A, affective; C, cognitive.*

#### Experimental setup

All physiological data were recorded with a 72-channel QuickAmp amplifier system (BrainProducts GmbH, Munich, Germany). EEG was measured using an actively shielded 64-channel electrode cap with Ag/AgCl electrodes which were arranged according to the international 10/10 system (ANT, Netherlands). The system was grounded at the participant’s shoulder. Data were recorded with a common average reference and filtered in a range from DC to 70 Hz at a sampling rate of 250 Hz and 22-bit resolution. For correction of eye movement and blink artifacts, a vertical and horizontal electrooculogram was measured by placing two electrodes above and below one eye and two electrodes on the left and right side of the eyes. Respiration rate was measured with a respiration belt and skin conductance on the second and third finger of the non-dominant hand. Additionally, an electrocardiogram (ECG) was assessed with two electrodes. In this report we only focus on the EEG data.

At least two experimenters were present. One of them (K.B.) served as instructor guiding the participants through the IAMEx. The other experimenter was monitoring the raw data during the recording and writing a time stamp protocol taking note of the start and end times, i.e., when the instruction was completed and when the next instruction was started. The participant was seated in a comfortable chair in one corner of the room. From there he/she could view a white and a green sheet of paper on the floor as well as a vase with flowers on a small board as specified in the IAMEx (see instructions above). All three objects were about 3–4 meters away from the participant and about 0.5–1.5 meters from each other.

### EEG DATA ANALYSIS

#### EEG Signal Processing

The whole data analysis was performed using Matlab. After detrending the DC recorded EEG data sets all EEG channels were corrected for eye movements using a linear correction algorithm which detects eye blinks and movement events and uses those periods to determine a correction factor for each channel. The electrooculogram was multiplied by this factor and then subtracted from the EEG according to Gratton et al. (1983).

A power spectrum time series was calculated using the fast Fourier transform (FFT). FFT was applied to the windowed EEG time series, which was convolved using a Nutall window and shifted in steps of 0.5 s. A window size of 2 s was chosen for calculation of the FFT frequency coefficients. This resulted in 140 FFT frequency bins from 0–70 Hz and a resolution of 0.5 Hz. To limit the influence of high-amplitude artifacts the spectral amplitudes were limited to 5 standard deviations. To obtain a measure of the power spectral density (PSD), FFT values were squared. The FFT bins were then averaged into 7 standard frequency bands: delta (1–3.5 Hz), theta (4–7.5 Hz), alpha1 (8–10 Hz), alpha2 (10.5–12 Hz), beta1, or SMR (12.5–15 Hz), beta2 (15.5–25 Hz), gamma (25.5–47 Hz), and an additional high gamma band (53–70 Hz). All data were visually inspected in a time-frequency-resolved fashion to detect periods of noise or bad signals in order to control for bad electrodes or longer periods of EMG noise or other artifacts. Short-term artifacts were controlled for by the use of medians as described below. The high gamma band was not expected to provide reliable information and therefore was only used for discussion of possible high-frequency artifacts such as muscle tension, which are normally more visible in the high frequencies. Thus, for statistical comparison only the first seven frequency bands were considered.

#### Epoching

Before segmentation of the data streams from the three recording sessions the PSD time trace was detrended in order to be independent from a possible sequence effect. Then, the data stream was cut into epochs according to the different task conditions, resulting in six resting state conditions (five with eyes open and one with eyes closed), one reading condition and two times the nine tasks of interest as shown in **Table 2**. For temporal averaging of the PSD time traces within each task epoch all 2 s intervals were averaged. As there were two intervals per second about 120 values were averaged in an epoch of about 1 min. In order to be robust against rare but possible high-amplitude artifacts the temporal median was used and the interquartile range served as a measure for the standard deviation which can be estimated by multiplication by 0.7413 (see Matlab function iqr). Thus, an average EEG PSD with its standard deviation was available for each task condition, electrode, frequency band and participant. Further reduction levels were achieved by averaging over all electrodes, resulting in the global band power.

In addition to the six single tasks for the analysis of locations and foci, three conditions were added with specific location only and no specific focus, i.e., both foci were merged into one epoch. Also, to analyze conditions that discriminate between the two foci only, two conditions were added in which all locations were merged. As shown in **Table 3** the unspecific or arbitrary location was indexed with an X as the first letter and the arbitrary focus was indexed with an X as the second letter. This resulted in 11 mental conditions of potential interest.

**Table 3:** A matrix showing the indices for 11 conditions of various foci and locations.

Mental location	Attentional focus		
	Self	Object	Both merged
Intrapersonal	IS	IF	IX
Extrapersonal	OS	OF	OX
Empathic	ES	EF	EX
All merged	XS	XF	–

*Location: I, intrapersonal; O, extrapersonal; E, empathic connection; X, all three locations were merged.*

*Focus: S, self or own physical sensations; F, flowers; X, both foci were merged.*

#### Statistical comparisons

In order to uncover the specificities of each condition it was necessary to contrast them with each other. According to our model in **Figure 1** two general types of comparisons can be distinguished, namely those comparing different locations with each other and those comparing the different foci. The location comparisons aim to distinguish between an internal, external and empathic mental position. These comparisons were made with an unspecific focus and the self- and object-oriented focus, resulting in nine comparisons [**Table 4(1)**]. The distinction between the object- and self-directed mental focus was calculated for merged mental locations and for the three specific locations [**Table 4(2)**]. An additional contrast condition was chosen which should show the difference between a “selfless” state, defined as an outside position with the attention directed toward an external object, and a self-centered state, defined as an internal mental position with self-directed awareness. The corresponding category was termed “relatedness” [**Table 4(3)**]. Finally, as some of the instructions require cognitive processes and others ask for affective and emotional involvement we decided to contrast the cognitive and affective processing modes by averaging all cognitive tasks (i.e., OS, OF, EF) and all affective tasks [i.e., IS, IF, ES; **Table 4(4)**]. Together with one additional comparison of the resting state with eyes open versus closed a list of 16 comparisons or contrasts of interest was available. As a measure of the difference between tasks within each person we chose the estimated effect size by using the formalism of Cohen’s d, however, we used the median of epochs instead of the mean and an estimated standard deviation using the interquartile range.

**Table 4:** The contrasted conditions are listed systematically.

		*Foci*			
		*Arbitrary*	*Self*	*Object*
(1) Comparisons of locations	*Intra-extrapersonal*	IX-OX	IS-OS	IF-OF	
	*Intrapersonal-empathic*	IX-EX	IS-ES	IF-EF	
	*Empathic-extrapersonal*	EX-OX	ES-OS	EF-OF	

			***Locations***		
		
		***All locations***	***Inside***	***Outside***	***Empathic***

(2) Comparisons of foci	*Object-self*	XF–XS	IF–IS	OF–OS	EF–ES

(3) Relatedness	*“Selfless”-self-centered*		OF-IS		

(4) Processing mode	*Cognitive-affective*	C–A = (OS + OF + EF)–(IS + IF + ES)			

*The comparisons in bold are those of special interest which formed the basis of our hypotheses.*

In order to account for the differences in the fulfillment of specific tasks the effect size of each participant, task, electrode, and band was multiplied by a self-rating factor specific for each task and participant. This self-rating factor was calculated by averaging the self-ratings (1–5) over all single tasks which formed the task condition and dividing by the average across all task conditions.

Before calculation of the effect across participants the Anderson–Darling test of normal distribution was performed, showing that delta and alpha values in particular were not normally distributed. As a consequence, a Wilcoxon signed rank test was applied to the differences in effects corrected for individual task fulfillment to estimate the significances of those task comparisons.

#### Further considerations

**FIGURE 2:**
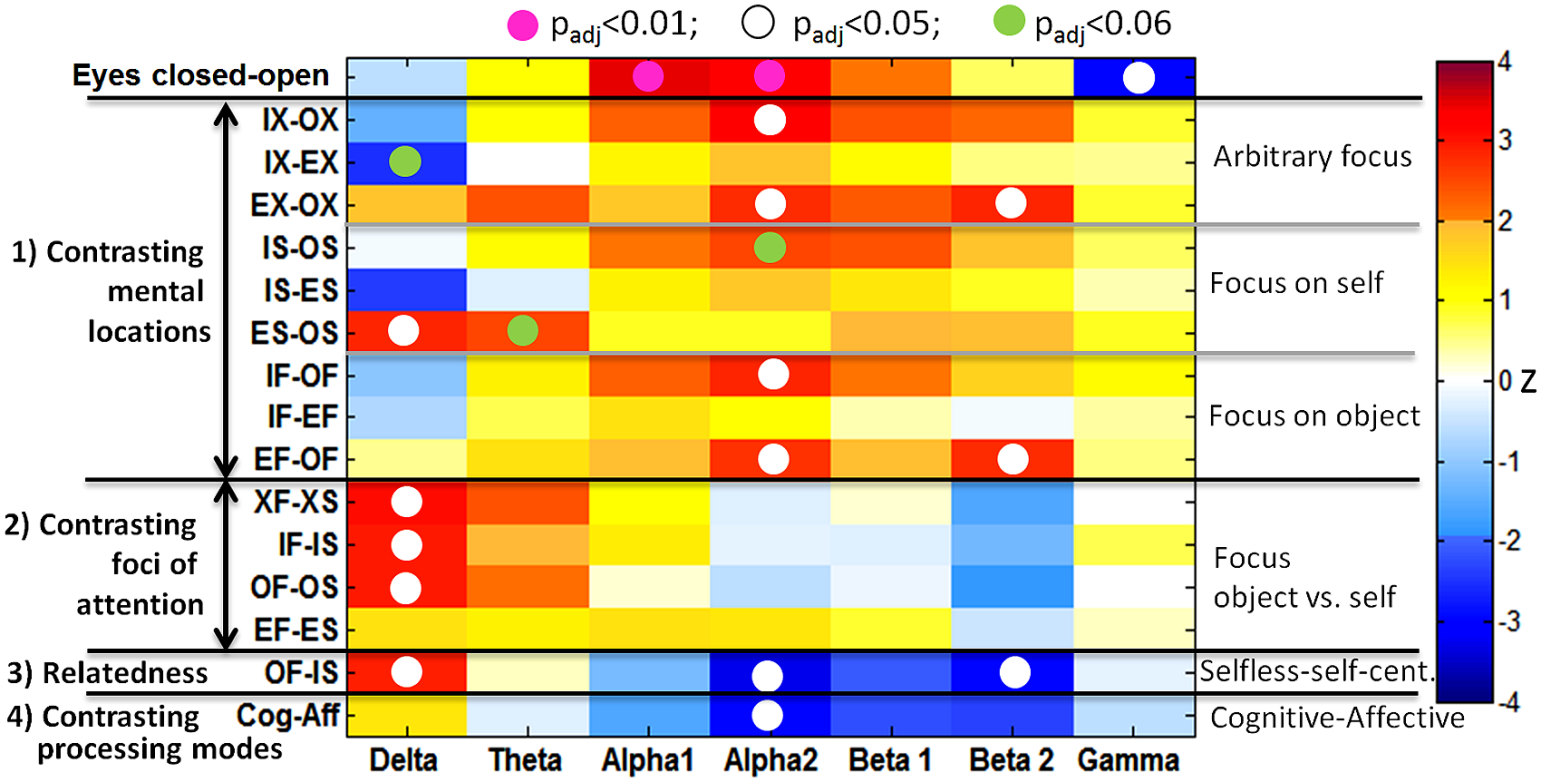
**Significances of mean differences between conditions displayed for the global band power measure**. Significant contrasts are shown as red and dark-blue fields. The white dots indicate that the *z*-score remained significant (*p* < 0.05) after FDR adjustment across seven frequency bands and 16 comparisons. Pink dots were significant at *p* < 0.01, and the green dots indicate *p* < 0.06.

**FIGURE 3:**
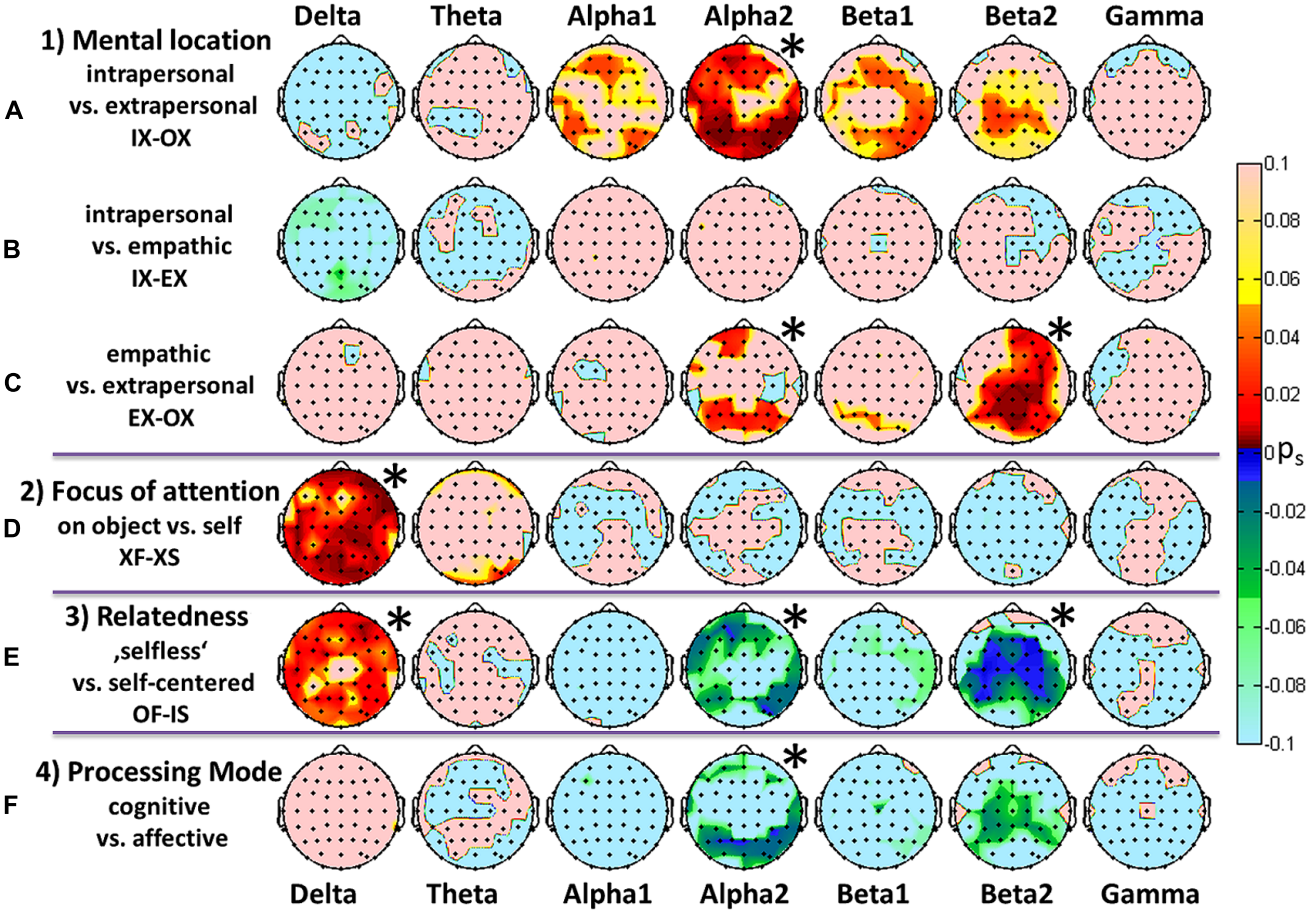
**Comparisons of the most prominent findings depicted as topographic color maps for all frequency bands**. FDR-adjusted signed *p*-values resulting from a Wilcoxon signed rank test for 16 participants. Red areas represent a significant increase while green/blue signaling represents a significant decrease in spectral power. **(A–C)** Different mental locations with an arbitrary focus. **(D)** Comparison of attentional foci, i.e., the flowers versus self-directed, independent of the location. **(E)** Differences between a “selfless” and a self-centered mind set reflected by a change in both position and focus. **(F)** Contrast between mental processing modes. The asterisks indicate overall significance according to **Figure 2**.

Correction for multiple comparisons is not trivial as such measures are highly dependent on each other. Therefore, Bonferroni correction of significance levels would be far too conservative and wipe out most effects. The false discovery rate (FDR) adjustment method constitutes a less conservative approach. Based on the formulas of Benjamini and Hochberg (1995) and Yekutieli and Benjamini (1999), we applied FDR adjustment to the *p*-values across six frequency bands and 15 comparisons. In the results shown in **Figure 2**, all values that survived FDR adjustment at the 5% level were marked with a white dot. A pink dot marked those values with *p* < 0.01 and a green dot was used to mark marginally significant results with *p* < 0.06. Thus, the topographic mapping in **Figure 3** of the dotted fields from **Figure 2** might present reliable positive results.

### HYPOTHESES

(H1) On the global scale, we expected band power differences in the EEG comparing all mental tasks as shown in **Table 4**. Topographic differences were calculated for the specific comparisons shown in bold in **Table 4**. Here, we assumed that the distinct subjective states of consciousness would express themselves in discriminable topographic and spectral EEG patterns. Specifically, we hypothesized that
(H2) Different *mental locations* would present different spectral EEG patterns. Therefore, the tasks IX, OX, and EX have to be compared to each other. According to Knyazev (2013), self-referential processing should show enhanced alpha and beta oscillations and diminished theta and delta activities. The same prediction could be made when hypothesizing that OX tasks reduce DMN activity, which is active in IX tasks according to Jann et al. (2010).
  The EX mode involves memory-related tasks because of the need to imagine a well-known person and therefore should exhibit increased theta waves (Ward, 2003).
(H3) Self-directed and object-directed *mental foci* should show different EEG patterns. The tasks XF and XS will be contrasted. According to Knyazev et al. (2011) extraversion (possibly similar to XF) could be predicted by increased posterior and decreased orbitofrontal theta activities. As self-referential thinking is sometimes associated with the DMN of the brain it is further hypothesized that the corresponding EEG characteristics will match those of the XS state.
(H4) *Self and other:* this is a special combination of the previous modes in which a self-concept is not involved at all and which is expected to be different to a state in which a self-concept is referenced to itself (OF-IS). Therefore, we would expect to find both the correlates of IX-OX (hypothesis 1) and the correlates of XF-XS (hypothesis 2) in this mode.
  Here also, according to Knyazev (2013), during self-referential processing alpha and beta oscillations should increase, while theta and delta activities should be larger in the “selfless” state (Knyazev, 2012).
(H5) As can be seen from **Table 1** we ask whether the IAMEx also presents a discriminable difference between *cognitive* and *affective processing* modes. This is an additional hypothesis which does not directly relate to the model of focus and location but is included because of the categorization of the modes into more affective and more cognitive ones. While cognitive processing is expected to show decreased alpha and increased theta band activities, emotional processing would exhibit increased alpha and right frontal gamma band oscillations.
(H6) The confounding variables age and gender should not have a significant influence on the results.

## RESULTS

### PARTICIPANT CHARACTERISTICS

The results of the questionnaires were used to characterize the sample of participants. Therefore, the participants’ responses to the FMI, BPS-20, and IAMI were compared with the population means taken from validation studies. Validation of the BPS-20 and IAMI included 1,089 participants, and the SCL-90R was compared with the population means published by Franke (2002). The results listed in **Table 5** reveal our participants as having higher personal boundary protection compared to the population mean (*d* = -1.29) and slightly higher individual attention management (*d* = 0.46); in particular, inner boundary management exceeded that of the normal population (*d* = 0.79). The participants also showed higher mindfulness scores (*d* = 0.45–0.72, depending on the factor). Psychopathological symptoms measured with the SCL-90R were within the range of the normal population (±1 SD) in 14 of 16 participants. Altogether, our participants formed a psychologically healthy sample with a high mindfulness self-rating and increased boundary protection as well as increased attention management abilities.

**Table 5:** Results of the BPS-20, IAMI, FMI, and SCL-90R questionnaires from participants in the current study compared with the population mean.

Inventory	(Sub-) Scale	Study mean (±Std)	Population mean (±Std)*	Effect size (Cohen’s d)
BPS-20	BPS	51.6 (±10.1)	62.2 (±8.2)	–1.29
IAMI	Total	186.0 (±19.0)	178.0 (±17.2)	0.46
	Inside	57.6 (±4.9)	52.5 (±6.5)	0.79
	Outside	77.9 (±8.4)	74.9 (±8.1)	0.37
	Empathic	50.4(±9.0)	50.6 (±7.9)	-0.03
FMI	Total	42.4 (±4.0)	37.7 (±6.4)	0.62
	presence	19.0(±1.6)	16.6 (±2.9)	0.72
	acceptance	23.4 (±3.0)	21.1 (±4.2)	0.45
SCL-90R	GSI	49.6 (±8.3)	50 (±10)	-0.03
	PSDI	46.9 (±8.1)	50 (±10)	-0.24
	PST	50.3(±8.1)	50 (±10)	0.02

*N = 1,089 (see Blaser et al.,2014a,b). GSI, Global Severity Index; PSDI, Positive Symptom Distress Index; PST, Positive Symptom Total; STD, StandardDeviation.

#### Task fulfillment

The fulfillment of each task was rated by the participants on a scale from 1 to 5. On average, 6.8 of the 16 participants rated the tasks as having been fulfilled with a score of 4.5 or 5; 6.1 of 16 gave a score of 3.5 or 4; 2.5 of 16 gave a score of 2.5 or 3, and 0.6 of 16 rated task fulfillment with low scores of 1–2. A detailed list for specific tasks is shown in **Table 6**.

**Table 6:** The average number of the 16 participants with the self-ratings for each task is listed.

Task	Fulfillment self-rating					
	*R* ≤ 2	*R* = 2.5 or 3	*R* = 3.5 or 4	*R* = 4.5 or 5
IS	0	1.5	5	9.5
IF	0.5	1	6.5	8
OS	1.5	3	6	5.5
OF	0	4	6.5	5.5
EF	0	2.5	7	6.5
ES	0	3	5.5	7
IS	2	2,5	6	5.5

### GLOBAL EEG DIFFERENCES

#### General effects

To generally test the differences between the 11 conditions across participants a non-parametrical Friedman test was applied to the spectral data of the 16 participants. Electrodes were used as blocking factor. All bands presented a highly significant effect with *F* > 73 and *p* < 0.001 except for the gamma band, which was not significant. Similarly, a Friedman test was applied to the effect sizes of the 16 comparisons between tasks with electrodes as blocking factor. All bands presented highly significant effects (*F* > 400, *p* > 0.001). This allowed for a detailed analysis, and, moreover, our global hypothesis (H1) was confirmed.

#### Specific global analysis

Significance values of PSD differences on a global scale (global band power) of 16 comparisons resulting from a Wilcoxon signed rank test with subsequent FDR adjustment of *p*-values across seven bands and 16 comparisons are illustrated in **Figure 2**. The first row shows the trivial result of an “eyes closed vs. open” contrast leading to a highly significant alpha1 (*z* = 3.46, *p*_adj_ = 0.007) and alpha2 (*z* = 3.36, *p*_adj_ = 0.009) power increase in closed eyes with a simultaneous gamma decrease (*z* = -2.95, *p*_adj_ = 0.02). This result will not be discussed further. In all other comparisons participants had their eyes open.

***Mental locations.*** Contrasting all inside with all outside mental locations showed a significant increase in alpha2 (*z* = 3.21, *p*_adj_ = 0.012), but also in the beta band, without reaching the significance level of 0.05 after FDR adjustment. Specifically, this alpha2 increase reached significance in the object-directed contrast IF-OF (*z* = 2.84, *p*_adj_ = 0.021). There was no difference in delta power between IS and OS, but it was significantly stronger in empathic self-perception compared to cognitive self-perception (ES-OS: *z* = 2.84, *p*_adj_ = 0.021). The hypothesis (H2) of increased alpha2 was confirmed, while the expected increase in beta activity did not reach significance after FDR adjustment, and decreased delta and theta could not be confirmed.

Increased alpha2 (*z* = 3.67, *p*_adj_ = 0.02) and beta band power, with a predominance of beta2 (*z* = 2.79, *p*_adj_ = 0.023), was visible in empathic connections with another person compared to the extrapersonal location (EX-OX). This effect was especially strong in the object-oriented focus EF-OF, with significantly increased alpha2 (*z* = 2.69, *p*_adj_ = 0.034) and beta2 (*z* = 2.79, *p*_adj_ = 0.023). The hypothesized increase in theta did not reach significance.

*Attentional foci.* Contrasting attentional foci revealed a different picture, with the strongest and highly significant differences in the delta band for the comparisons XF-XS (*z* = 3.10, *p*_adj_ = 0.017), IF-IS (*z* = 2.95, *p*_adj_ = 0.019) and OF-OS (*z* = 3.00, *p*_adj_ = 0.019). This would confirm the hypothesis (H3) of an increase in delta in the externally directed foci, which would reduce DMN activity. The change in the theta band did not reach significance after FDR adjustment, and the alpha and beta bands were not sensitive to the attentional focus as hypothesized.

*Relatedness.* Taking both comparisons together, i.e., foci and locations, constitutes the category relatedness. Here, an outside mental location with the focus on an external object (OF) showed increased delta band activity (*z* = 2.90, *p*_adj_ = 0.021) and decreased alpha2 (*z* = -3.26, *p*_adj_ = 0.011) and predominantly beta2 activity (*z* = -2.95, *p*_adj_ = 0.019) in contrast to an internal self-focused mental awareness (IS), as shown in the second last row of **Figure 2**. As suggested in hypothesis H4 we found a combination of the correlates of IX-OX and XF-XS with significant patterns in delta, alpha2 and beta2 bands.

***Processing mode.*** Clear similarities were visible between the categories relatedness and the processing mode. In the latter, only the alpha2 band reached significance after correction (*z* = -2.95, *p*_adj_ = 0.019). The alpha decrease in cognitive modes could be confirmed, while a theta increase in cognitive modes and higher gamma band activities in emotional processing, as proposed in hypothesis H5, could not be found.

#### Influence of age and gender

Although the statistical power with 6 male subjects seems to be quite small for detecting reliable gender differences we tested for possible differences. A Kruskal–Wallis test on the global band power differences across 16 comparisons and 7 frequency bands did not show significant differences between male and female participants [χ^2^(*df* = 1) = 0.6, *p* = 0.44]. A separate analysis of each frequency band also did not reveal significant gender differences.

An ANCOVA with the confounding factor age was performed on the effect sizes of power differences between tasks for 16 comparisons and seven bands. The findings show that the overall result remains significant after considering age as a confounding factor (*F* = 5.42, *p* = 0.02). A correlation analysis using Spearman’s rank correlation between age and the effects of 16 comparisons and seven frequency bands revealed no significant correlations after FDR adjustment. Therefore, it can be concluded that age does not play a significant role in this context and hypothesis H6 holds true.

### TOPOGRAPHIC DIFFERENCES

**Figure 3** displays topographic mappings from the three general comparisons between the three mental locations (3a–c), the general comparison of the object- versus self-directed focus (3d), the “selfless” versus self-centered states (3e) and the contrast of cognitive and affective processing (3f). A Wilcoxon signed rank test with subsequent FDR adjustment of *p*-values at the level of electrodes was calculated on the effects weighted with the fulfillment self-rating. The resulting *p*-values were multiplied by the sign of the corresponding *z*-values. The topographic maps thus show the significant signed *p*-values.

#### Mental locations

Comparisons of intrapersonal and extrapersonal tasks presented highly significant increases in the parieto-occipital and midfrontal and prefrontal alpha and low beta PSD. In contrast, the high beta activity revealed a significant increase in the medial-parietal region. Other frequency bands did not show any noteworthy changes. Similar but much weaker increases could be observed when contrasting empathic connectedness and the extrapersonal location for those three bands. In the high beta band the increase was shifted toward right centro-parietal regions.

#### Attentional foci

Completely different spectral and topographic patterns resulted from the comparisons between different foci of attention. Here, significant changes could be observed almost globally in the delta band only.

#### Relatedness

The alteration of mental location and attentional focus in the comparison OF-IS led to the strongest differences in PSD. A global increase in delta activity was accompanied by a decrease in the frontal and lateralized temporal and parietal alpha2 band, as well as a highly significant lateralized beta2 decrease.

#### Processing modes

States which required cognitive processing showed decreased activities in the posterior alpha2 and centroparietal beta2 bands.

## DISCUSSION

Our results indicate that both mental locations and attentional foci showed significant characteristics in neurophysiological data measured by 64 channels of EEG. Generally, we found that the alpha2 and beta2 bands served as good indicators for (a) the distinction between intrapersonal (IX) and extrapersonal space (OX), (b) the distinction between perspective taking (empathy; (EX) and extrapersonal space (OX), (c) the distinction between “selfless” and self-centered states, and (d) the distinction between affective and cognitive processing modes. The topographic similarities suggest that those four polar categories seem to be represented by similar neural mechanisms. In contrast, the delta band served as an indicator for the distinction of attentional foci (object vs. self). In the following it is attempted to use the results for a clearer characterization of the concepts “intrapersonal,” “extrapersonal,” “empathic,” “self-centered,” “selfless,” “cognitive processing,” “affective processing,” and “focus of attention.”

### Mental Locations

#### Intrapersonal (IX in **Table 3**)

According to the instructions in the IAMEx, both the IS and IF states represent a mindful, affective or emotional perception of either a person’s own body or the environment. The intrapersonal space therefore represents a first-person perspective. In contrast to the extrapersonal space we found significantly higher alpha2 band power over frontal brain areas and lateralized parietal areas. According to Vogeley and Fink (2003) the medial prefrontal, medial parietal and lateral temporoparietal cortex is involved in the first-person perspective. The medial parietal cortex is related to the viewpoint of the observing self. This may be supported by our findings for the beta2 band. The results also fit with the findings of Knyazev (2013), who reported enhanced alpha oscillations during self-referential cognitive processing and enhanced beta activity in the postcentral gyrus while theta and delta activities were reduced in the superior frontal gyrus. However, we did not find any reduction in theta activity.

Higher left parietal alpha was also reported by Mu et al. (2008) during pain empathy compared to processing of neutral stimuli. Only small and non-significant differences in the empathic states (IF-EF) and the self-directed state were visible. Therefore, the intrapersonal space could be interpreted as a self-experiencing or even “self-empathic” process through which the world is perceived. In considerable concordance with the highly significant lateralized centroparietal alpha2 effect, the experience of self-location and hand ownership has been found to be related to bilateral sensorimotor cortices and posterior parietal alpha increases as well (Lenggenhager et al., 2011; Evans and Blanke, 2013).

#### Extrapersonal (OX in **Table 3**)

The extrapersonal space represents a cognitive construct of the world. It can also be thought of as a dissociated state which, according to Damasio (1999), is characterized by an active inhibition of emotional activity in the medial prefrontal cortex, which is essential for monitoring and modulation of emotions. In addition, decreased power in frontal and parietal beta PSD (**Figures 3A,C**) supports this theory. Those regions seem to be less activated when subjects were located mentally in the extrapersonal space, which is a cognitive space. This is in line with the findings of Gusnard et al. (2001) and Raichle et al. (2001) showing those regions to be activated during resting states.

#### Perspective taking (empathy; EX in **Table 3**)

Taking over another person’s perspective is performed as a cognitive and an affective empathic connection. The affective empathic connection was found to be associated with activations in the inferior frontal gyrus (Shamay-Tsoory et al., 2009), anterior insular cortex and dorsal anterior cingulate cortex (Lamm and Singer, 2010). Unfortunately, subcortical activities are usually not visible on EEG. Lamm et al. (2007) associated activations in the right parietal cortex with the adoption of another person’s perspective. This is in line with our highly significant right parietal beta2 increase in the comparison of EX and OX. These EEG findings are also supported by Baars et al. (2003), who found heightened fMRI activity in medial parietal, inferior lateral parietal and prefrontal cortical areas when participants were asked to adopt the visual perspective of another person. Findings as reported by Lamm et al. (2007) in an fMRI study showing that the left parietal cortex demonstrated higher activity in the self-perspective, whereas the right parietal cortex was associated with the adoption of another’s perspective, could not be demonstrated with our method. The fact that often subcortical structures in the parietal cortex seem to distinguish between the perspective of the self and that of another person might explain the small and non-significant changes between IX and EX.

Sadaghiani et al. (2010) stated that alpha2 band activity was positively correlated with activations of the dorsal anterior cingulate cortex, anterior insula, anterior prefrontal cortex and thalamus. This is in line with the increased interoceptive awareness in IX, attributed in particular to the anterior insula. In a study by Foxe et al. (1998), enhanced alpha synchronization was also attributed to selective attention and was proposed to reflect disengaged anticipatory activities, while active anticipation reduced the alpha oscillations. This would indicate that the enhanced alpha activity in the intrapersonal location might suppress the flood of information that other perspectives would require, and the inside view would therefore be the simplest.

### FOCUS OF ATTENTION

#### Object- versus self-directed attention

With regard to the detection of EEG correlates of self-referential processes, the in-depth review of Knyazev (2013) mentions that delta and theta oscillations (most prominently in frontal midline regions) correlate negatively with activity in the DMN. We found highly significant global delta activations in the object-directed attention task, indicating inhibition of the DMN during XF tasks. According to Knyazev (2012) higher delta activity during “selfless” states suggest that the basic homeostatic and motivational processes are rather object-related states without the necessity of a self-construct. It seems important to note that alpha2 does not seem to be sensitive to the attentional focus but is strongly responsive to the mental location.

### RELATEDNESS

#### “Selfless” versus self-centered

As can be seen in **Figure 3**, the contrast of a “selfless” state, i.e., an object-oriented external perspective, and a “self-centered” state, i.e., a self-oriented intrapersonal perspective, displayed the most prominent differences in the delta, alpha2, and beta2 bands. The relatedness concept represents a combination of location and focus alterations, and in fact the physiological results also tend to show a combination of the results between IX-OX and XF-XS. This supports the idea that self-centeredness can be neurophysiologically separated into the aspect of intrapersonal mental connectedness and a self-directed attentional focus. The findings suggest that “selflessness” and self-centeredness might be intellectually and physiologically relevant concepts. Most of the differences are in line with the findings of other research groups. We found higher right frontotemporal delta power in “selfless” states compared to self-centered states. The OF state may also be related to a dissociated state. Dissociation has often been associated physiologically with the temporal lobe (Bob, 2003), which in our study showed increased slow waves and decreased fast frequencies compared to a self-centered state. The increased delta activity was accompanied by decreased bihemispheric frontal, central and parietal beta2 power, suggesting decreased processing in this region. Wheeler et al. (1997) reported that in some cases lesions in the right frontotemporal cortex led to the experience of cognitive detachment from the self. Interestingly, the contrast of “selfless” with self-centered shows a very similar picture, especially with regard to alpha and beta bands, to the contrast between “thoughtless emptiness” and a state of presence referred to as open monitoring as measured in a study with 30 meditators (Hinterberger et al., 2014). In fact, the state of being present in the moment with an awareness of the physical space of the body represents the IS state, while pure observation of an object from outside (the instruction in the OF state did not ask for cognitive thoughts about the object) might come close to a non-attached thoughtless state. The contrast between visual perception and self-reference is strongly visible in the left and right fronto-centro-parietal beta2 decreases. These lateralized effects could indicate visual information processing in the ventral stream, which is related to object recognition (Goodale and Milner, 1992; Brown, 2009). In contrast, an association between self-reference and activation in cortical midline structures (e.g., Northoff and Bermpohl, 2004) was not visible here.

### PROCESSING MODE

#### Affective versus cognitive processing

In psychology and neuroscience we find a distinction between cognitive and emotional or affective processing. Damasio (1999) has summarized the findings in relation to the mechanisms of affective processing. He describes emotions as the basis for the self and the self-model. Newen and Vogeley (2007) suggest that the neural correlates of the first-person perspective are associated with the medial prefrontal and parietal cortex and the temporo-parietal junction. These are deactivated when subjects perform cognitive tasks (Raichle et al., 2001) and could represent an indicator for affective versus cognitive mental states. The affective modes IS, IF, and ES require a sensorily driven sense of body ownership, which has been found to be associated with activations in midline cortical structures (Tsakiris et al., 2010). These modes present increased beta2 power in central areas, as visible in **Figure 3F**. Decreases in parietal and occipital alpha in cognitive modes become plausible due to the fact that cognitive modes require more visual information processing while self-perception promotes the visual alpha rhythm. Our findings for the alpha2 band are in line with the cognitive reduction as reported by Klimesch (1996, 1999) and Ramos et al. (1993). Ramos et al. (1993) also found the decreased beta during cognitive tasks. The affective enhancement of posterior alpha is in line with Uusberg et al. (2013). The right frontal gamma increase as reported by Müller et al. (1999) could not be observed. Despite this close link between affective processing and the self, the distinction between affective and cognitive modes as shown in **Figure 3F** presents similar effects, albeit not as strong, to the comparison between the “selfless” and self-centered mode. The delta contrast is also less pronounced. This raises the question whether the categories self-centered versus “selfless” processing are physiologically more pronounced in the EEG and possibly more relevant in terms of brain processing than the categories cognitive versus affective.

### TASK DIFFICULTY

As we were measuring objective physiological correlates of subjective mental tasks the validity of our findings depends on the actual performance of each task. We attempted to assess this individually for each task using a task fulfillment self-rating score. In this analysis we have decided to include the self-rating scores as a weighting factor in the effect sizes of each task, assuming that this would lead to results with a higher validity. Actually, the weighted results did not differ from the non-weighted results with regard to the essential findings.

The observation that the first two intrapersonal modes were more reliably achieved than the following modes supports the idea that these might reflect well-trained networks such as the DMN. Cognitive states and perspective taking might be harder to achieve as they require the performance of dual tasks, namely (a) the projection of one’s own viewpoint to an external place or to an external person and (b) answering questions about the attentional focus. The fact that the final IS mode was much more difficult to achieve than the initial one suggests that task fulfillment depends on the previous task. Gundel and Wilson (1992) showed that higher task difficulty resulted in the reduction of parietal and occipital alpha activity due to the amount of visual scanning as well as an increase in theta activity in the left frontal electrodes, which they hypothesize to be associated with the amount of general mental processing. This might further support the significant parietal and occipital alpha2 increase in the contrasts IX-OX and EX-OX because the OX tasks involve multitasking situations and were reported to be the most difficult ones and therefore are expected to show reduced alpha.

### LIMITATIONS OF THE STUDY

The sequence of the tasks was fixed, and therefore we do not know how well the EEG changes can be transferred to other sequences. It can be assumed that there is a significant carryover effect between tasks, which becomes visible when comparing the initial and final IS modes, as they were spectrally and topographically different. Therefore, these results may only be valid for the IAMEx sequence used here and may not be generalizable. For this, a further replication of this study concept with randomized task sequences would be necessary. Further, associations of EEG findings with fMRI results should be treated with care, and probably a LORETA analysis of our data would provide a more robust basis for such interpretations. For a replication of this study we would suggest the use of fMRI directly. Finally, the limited number of participants also calls into question the robustness of the results.

## CONCLUSION

We presented a spatial model of different forms of interpersonal perception, and our results confirm the neurophysiological discriminability of three mental locations and two attentional foci. The model of interpersonal attention management which served as the basis for this study seems to provide a useful concept for research in the domain of consciousness science. Different mental perspectives such as intrapersonal positions, cognitive and affective extrapersonal positions, and empathic connectedness were consciously occupied and could be related to specific EEG patterns.

It is likely that depressive patients and patients with a clear psychiatric diagnosis but also with psychotic symptoms such as hallucinogenic experiences differ in their ability to access these different mental positions and attentional foci. We further hypothesize that people with dissociative disorders or depressive episodes have difficulties with the free choice of and transition between mental locations. A further study with a clinical sample may therefore demonstrate that such patients present smaller contrasts between the tasks of the IAMEx. The IAMEx in combination with physiological measurements could therefore serve as a diagnostic tool. The IAMEx itself could serve as an exercise for psychosomatic rehabilitation. Further, the data suggest that via the presented exercises individuals could learn to generate brainwaves in specific frequency bands at will. This suggests the possible development of a neurofeedback device to train attentional and intra- and interpersonal flexibility for therapeutic and recreational purposes. Individuals who have difficulty empathizing with others could probably also profit from these methods.

We have presented data showing the neurophysiological distinction between concepts of intrapersonal versus extrapersonal mental space, “selfless” versus self-centered mental states, and cognitive versus affective processing. All three conceptual dualities showed very similar EEG PSD patterns in their comparisons. Although the category cognitive versus affective might be the most well-known differentiation in psychology it did not present the strongest effects. The most prominent differences, in the “selfless” versus self-centered contrast, showed a combination of the results in the location- and focus-dependent contrasts. This suggests that both the mental location and the attentional focus play a fundamental role in brain processes related to the self-concept and that they could be distinguished in the present study with respect to brain oscillations. Thus, the attentional focus and the so-called mental location seem to provide physiologically relevant categories because the contrast between intrapersonal and extrapersonal location displayed significantly different EEG patterns compared to the contrast between self- and object-directed attentional focus. At this point, we would like to raise the following philosophical question: what is the relationship between the mental concept of subjective experience and the concepts or terms we use to describe the organization and function of the brain? This seems to be an important question as our mental categories are essentially responsible for the interpretation of physiological findings and thus form our picture of the mechanisms of the brain.

## Conflict of Interest Statement

The authors declare that the research was conducted in the absence of any commercial or financial relationships that could be construed as a potential conflict of interest.

## Footnotes

1 These properties of mental location and attentional focus in a mental space might be metaphorically related to the physical properties position and momentum in the physical space-time model. This would imply that mental location and attentional focus are complementary mental categories.

2 We have used quotation marks to indicate that the term “selfless” should not be construed with its usual meaning of “altruistic.”
